# Identification of predictors of long‐term survival and prognostic outcomes in thymic squamous cell carcinoma: A real‐world study

**DOI:** 10.1002/cam4.6049

**Published:** 2023-06-17

**Authors:** Meng‐Xin Zhou, Ye‐Ye Chen, Cheng Huang, Lei Liu, Gui‐Ge Wang, Jia‐Qi Zhang, Wen‐Liang Bai, Ke Zhao, Shan‐Qing Li

**Affiliations:** ^1^ Department of Thoracic Surgery, Peking Union Medical College Hospital Chinese Academy of Medical Sciences & Peking Union Medical College Beijing China

**Keywords:** chemoradiotherapy, surgery, thymic squamous cell carcinoma, TNM system

## Abstract

**Background:**

Although thymic squamous cell carcinoma (TSCC) is among the most prevalent forms of thymic carcinoma, there are relatively few studies on this tumor type, and its staging, optimal treatment strategies, and relevant prognostic factors remain controversial.

**Methods:**

The present study analyzed 79 patients diagnosed with TSCC between January 2008 and January 2021. Kaplan–Meier curves and Cox univariate and multivariate regression analyses were used to explore factors associated with overall survival (OS) and progression‐free survival (PFS) in the overall patient cohort and patient subgroups stratified according to the TNM stage. Time‐dependent receiver operating characteristic (ROC) analyses were used to compare the TNM and Masaoka systems as predictors of patient prognosis.

**Results:**

The 5‐ and 10‐year OS rates in this study were 65.5% and 49.4%, respectively, with corresponding 5‐ and 10‐year PFS rates of 52.3% and 37.9%. Survival outcomes were better for patients with early‐stage disease (*p* < 0.001) and patients that underwent surgical treatment (*p* < 0.001). Neither extent of resection (*p* = 0.820) nor the surgical approach (*p* = 0.444) influenced patient survival. In individuals with advanced disease, all forms of adjuvant therapy including radiotherapy (*p* = 0.021), chemotherapy (*p* = 0.035), and chemoradiation (*p* = 0.01) significantly improved patient PFS, but only adjuvant chemoradiotherapy improved patient OS (*p* = 0.035). When predicting the patient survival outcomes, the TNM system was slightly superior to the Masaoka system (area under the ROC curve [AUC] at 5 years: OS, 0.742 vs. 0.723; PFS, 0.846 vs. 0.816).

**Conclusion:**

TSCC is an orphan malignancy with a poor prognosis. TNM staging may be superior to Masaoka staging as a predictor of TSCC patient prognosis. Surgery is the mainstay of TSCC treatment. Video‐assisted thoracoscopy (VATS) should be considered for selected patients. Multimodal therapy was associated with excellent results for patients with advanced TNM stage, particularly when surgery was accompanied by adjuvant chemoradiation.

## 1 INTRODUCTION

Thymic epithelial tumors (TETs) are rare malignancies with an estimated incidence rate of 1.7–3.2 per million person‐years.^1^, ^2^ These tumors are stratified into the thymoma and thymic carcinoma subtypes, with the latter typically being more aggressive and correlated with a poorer prognosis.^3^, ^4^

Thymic squamous cell carcinoma (TSCC) is the most common subtype of thymic carcinoma,^5^, ^6^, ^7^, ^8^ accounting for 73.4%–80% of thymic carcinoma cases.^6^, ^8^, ^9^, ^10^, ^11^, ^12^ Earlier studies have tended to analyze thymic carcinoma cases as a unified whole without considering the different pathological subtypes. This has the potential of inducing bias in the findings due to differences in the characteristics of the subtypes, resulting in misleading study conclusions.^13^ We are only aware of three large‐scale studies that have examined the optimal treatments and prognostic factors associated with TSCC.^8^, ^12^, ^14^ The results of these studies were inconsistent, leading to difficulties in clinical decision‐making.

The present study aimed to provide more up‐to‐date real‐world evidence regarding the characteristics and prognosis of TSCC patients, through the analysis of 79 TSCC patients from a single Chinese institution to identify predictors of overall survival (OS) and progression‐free survival (PFS).

## 2 METHODS

### 2.1 Patients and materials

This was a retrospective, single‐center, observational study of patients admitted to our hospital with a pathologically confirmed diagnosis of TSCC between January 1, 2008 and January 1, 2021. The study was terminated on August 1, 2021, allowing a follow‐up period of over 6 months.

The inclusion criterion was that all patients had our hospital's histopathological diagnosis of TSCC. The exclusion criteria included: (1) the diagnosis was indefinite or made by another hospital, (2) the clinical data were incomplete, and (3) patients were lost to follow‐up.

The clinical data for the patients included age, sex, year of diagnosis, concurrent diseases, tumor size, disease stage, treatments, and follow‐up status. We divided the year of diagnosis into two groups from the middle to evaluate differences between surgical approaches used at different times. Tumor size was defined as the maximum tumor diameter measured via chest computed tomography (CT) imaging prior to treatment.^15^ Direct tumor size measurements were not utilized, as not all patients underwent surgery, and tumor resection was incomplete in certain patients that did undergo surgery. Disease staging was based upon the 8th edition of the AJCC TNM staging system^16^ and the Masaoka system.^17^ Disease staging was independently confirmed by two thoracic surgeons (Z. MX and C. YY), who reviewed patient medical records and pathological reports. Any discrepancies were resolved through consultation with a third expert (L. SQ).

Subgroup analyses were performed by separating patients that had undergone surgery into early‐stage (TNM stage I/II) and advanced‐stage (TNM stage III/IV) groups for a more reliable assessment of the relationship between outcomes and treatment modalities. The factors included the extent of tumor resection (complete vs. incomplete), the selected surgical approach (thoracotomy vs. video‐assisted thoracoscopy [VATS]), and postoperative adjuvant therapy (chemotherapy alone, radiotherapy alone, or chemoradiotherapy).

OS was defined as the interval between biopsy or surgery and all‐cause death or the most recent follow‐up. PFS was defined as the interval between biopsy or surgery and documented disease progression (including tumor recurrence, enlargement, and metastasis) or death without progression. Outpatient records and telephone calls were used to assess patient follow‐up status. Those patients alive at the last follow‐up were censored.

The study was approved by the institutional review board of our hospital (ZS‐3305). All patients provided written informed consent for the research use of their data at the time of hospital admission, and the study was thus exempt from the further need to obtain informed patient consent.

### 2.2 Statistical analysis

Normally distributed continuous data are presented as means ± SD and were compared via Student's *t*‐tests. In contrast, non‐normally distributed continuous data are presented as median (range) and were compared via Mann–Whitney U‐tests. For survival analyses, median values were used to transform non‐normally distributed continuous variables into dichotomous variables. Categorical variables were analyzed using chi‐squared tests or Fisher's exact test. Spearman correlation analyses were used to assess relationships between TNM stage and tumor size. The prognostic utility of the TNM and Masaoka staging systems was assessed using the time‐dependent receiver operating characteristic (ROC) curves, and the area under the curve (AUC) values at 1, 3, and 5 years were compared.

Kaplan–Meier curves were used to evaluate patient OS and PFS. Prognostic variables were identified through Cox proportional hazards regression models, with variables exhibiting *p*‐value <0.2 in the univariate analyses incorporated into the subsequent multivariate analyses.

R version 3.6.3 (The R Foundation for Statistical Computing) was used for all data analysis. The R packages timeROC, ggplot2, and survminer were used for evaluating survival. Two‐tailed *p*‐values <0.05 were considered statistically significant.

## 3 RESULTS

### 3.1 Clinical characteristics

**FIGURE 1:**
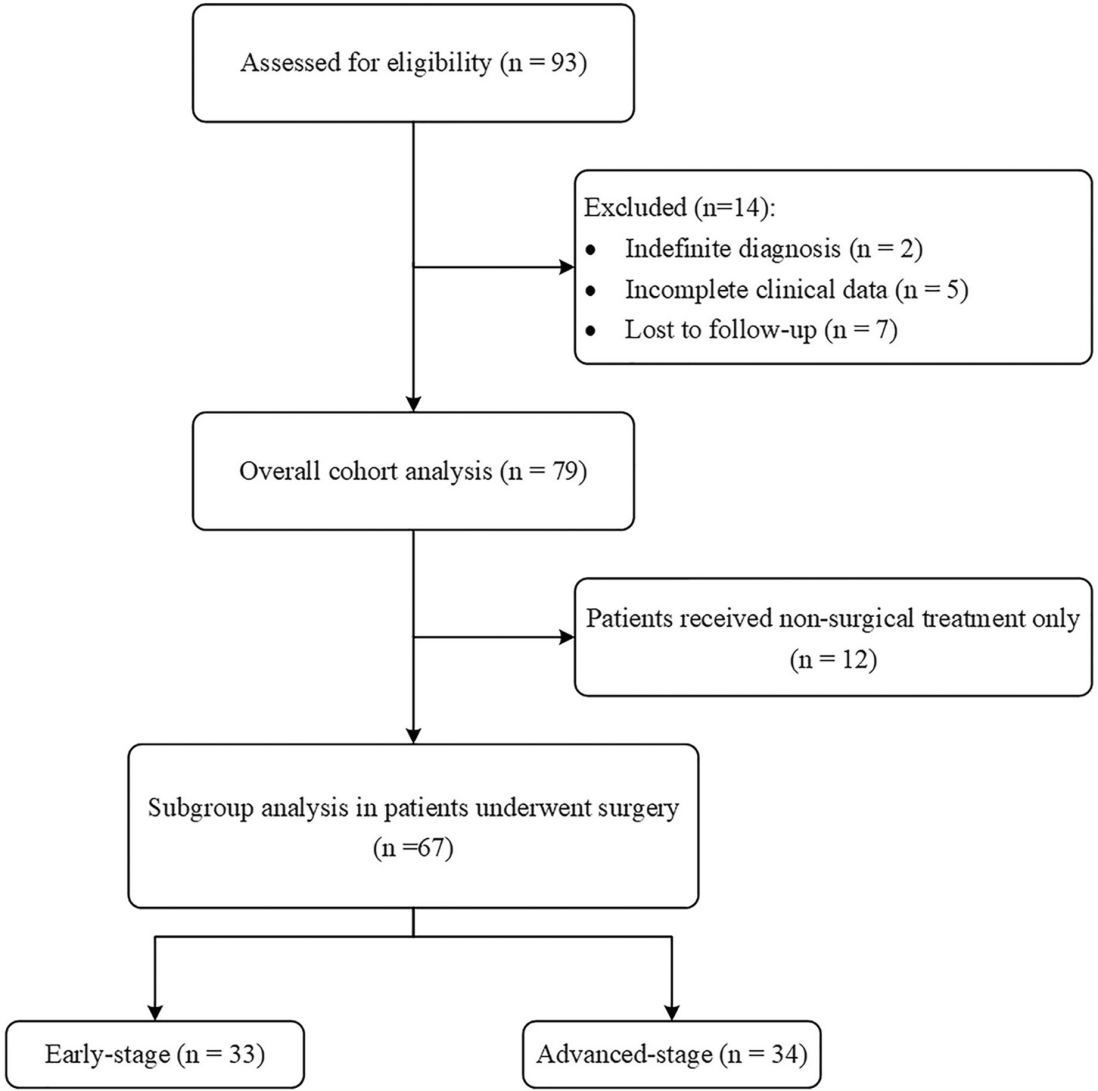
Flow diagram for patient selection.

**FIGURE 2:**
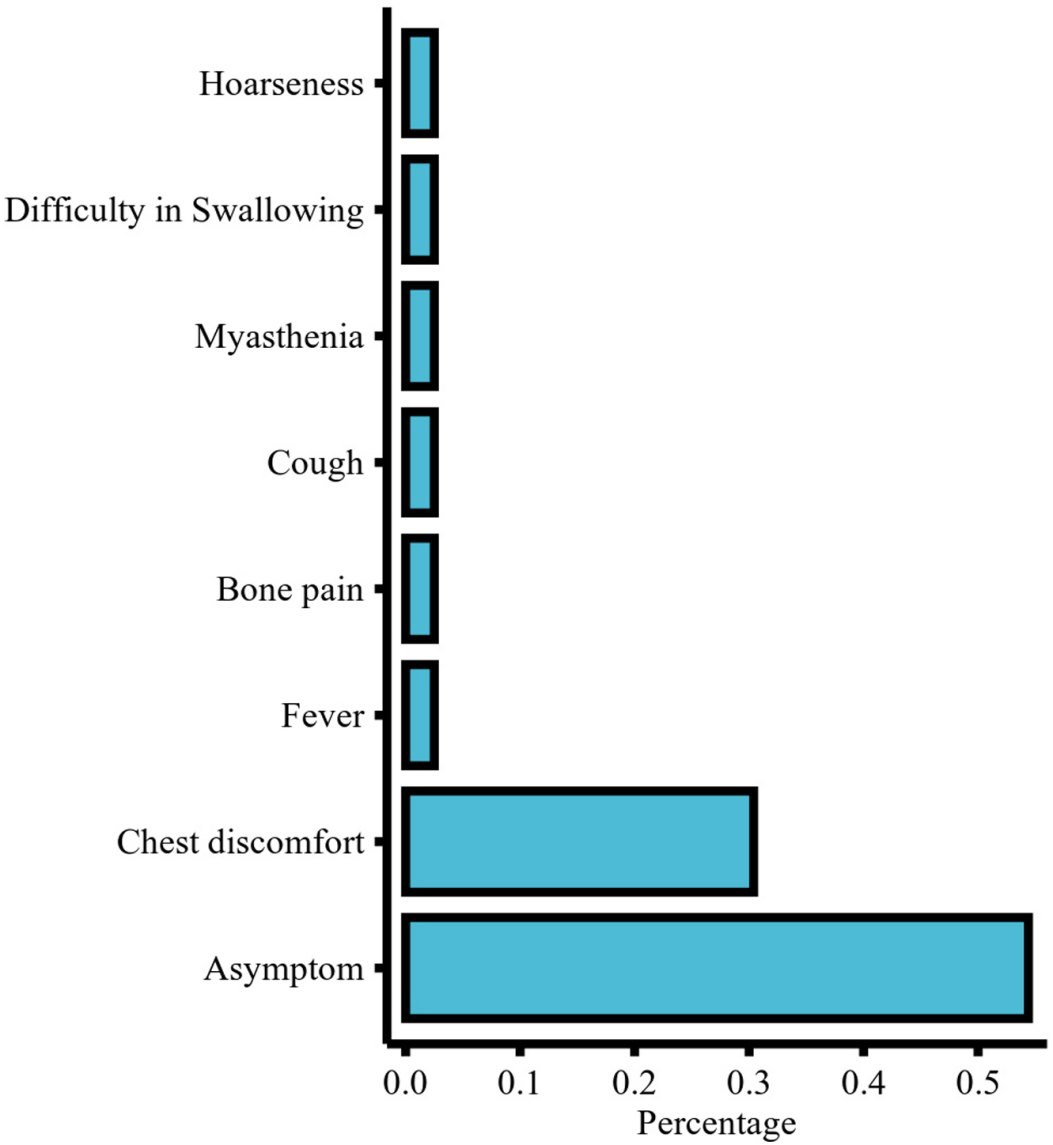
TSCC patient symptoms.

In total, 91 TSCC consecutive patients who were admitted to our hospital over the study period were initially recruited. Twelve of these patients (13.2%) were lost to follow‐up. The study thus included the remaining 79 patients (Figure 1). Their clinical characteristics are summarized in Table 1. The male‐to‐female ratio was 1.47:1, with an average age of 54.7 ± 10.6 years. The number of cases detected between 2008 and 2014 was comparable to the number of cases detected between 2015 and 2020. The most prevalent symptoms included chest discomfort, such as chest pain and chest tightness, which were experienced by more than 30% of the patients (Figure 2). Forty patients (50.6%) suffered from concurrent diseases, of which hypertension was the most common, occurring in 21 patients (26.6%). Myasthenia gravis (MG) was only observed in 3 patients (3.8%). The treatment administered, namely, surgery (*p* = 0.962), chemotherapy (*p* = 0.721), and radiation (*p* = 0.678) did not differ significantly between patients with and without concurrent disease. The median tumor size was 4.6 cm, and tumor size was moderately correlated with the patient TNM stage (*ρ* = 0.521, *p* < 0.001).

**TABLE 1:** TSCC patient clinical characteristics

Features	Number (%)
Age, years	
Mean ± SD	54.7 ± 10.6
Sex	
Male	47 (59.5%)
Female	32 (40.5%)
Year of diagnosis	
2008–2014	40 (50.6%)
2015–2020	39 (49.4%)
Tumor size, cm	
Median (range)	4.6 (1.5–12.0)
AJCC TNM stage	
I	26 (32.9%)
II	7 (8.9%)
III	22 (27.8%)
IV	24 (30.4%)
Masaoka stage	
I	16 (20.3%)
II	12 (15.2%)
III	27 (34.2%)
IV	24 (30.4%)
Treatment modality	
Surgery only	6 (7.6%)
Chemotherapy and/or radiotherapy	12 (15.2%)
Surgery plus adjuvant therapy	61 (77.2%)
Chemotherapy	12 (15.2%)
Radiotherapy	29 (36.7%)
Chemoradiation	20 (25.3%)

**FIGURE 3:**
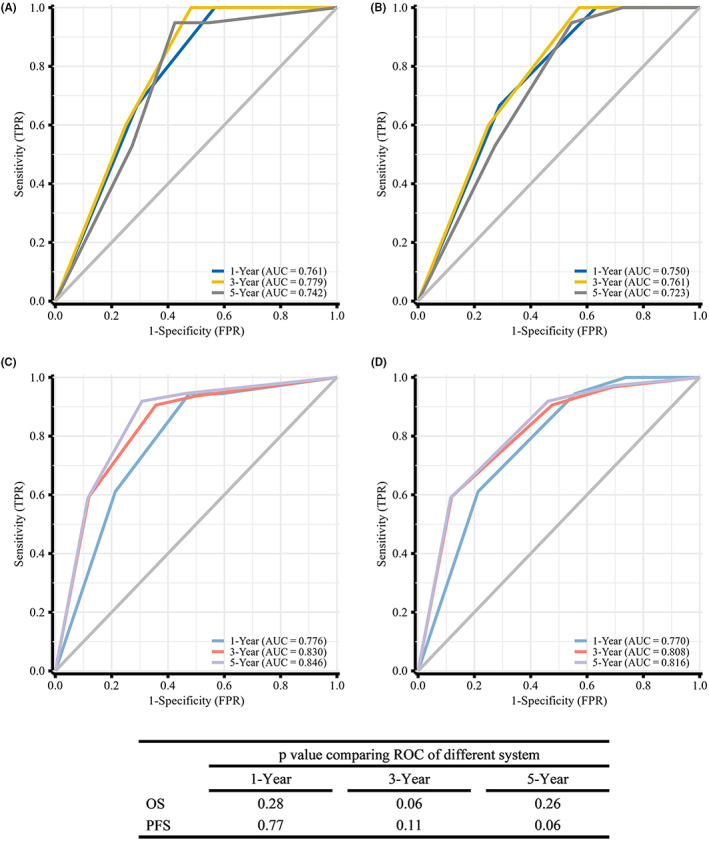
Time‐dependent ROC analysis of the TNM system (A) OS; (C) PFS and the Masaoka system (B) OS; (D) PFS.

As per the Masaoka system, 64.6% of the included patients had stage III/IV disease, while according to the TNM system 58.2% of patients had advanced‐stage disease. The ROC curves for these two staging systems and corresponding 1‐, 3‐, and 5‐year AUC values are shown in Figure 3.

The main surgical indications were as follows: (1) anterior mediastinal mass with suspected diagnosis of malignant tumor; (2) two or more thoracic surgeons had considered that the mass was resectable; (3) whole body examination indicated that there were no multiple systematic metastases; (4) debulking surgery was performed to control symptoms. The surgical approach was decided by the thoracic surgeon in charge. Overall, 67 patients (84.8%) underwent surgical treatment, of whom 58.2% underwent thoracotomy. VATS was the most commonly performed procedure between 2015 and 2020 relative to 2008–2014 (61.8% vs. 21.2%, *p* < 0.001).

Complete resection was unsuccessful in one patient among those with early‐stage disease. The tumor had invaded the pericardium and the final pathological result showed positive microscopic margins in the resected pericardium. Among patients with advanced‐stage disease, 12 underwent partial pneumonectomy and 6 vascular reconstructions. Pleural disseminations were observed in five patients during the surgery, resulting in incomplete resection. Debulking surgery aimed at symptom alleviation was performed on five patients.

Table 2 summarizes the differences in clinical characteristics between patients with early and advanced disease. Patients with early‐stage TSCC were more likely to have smaller tumors and to have undergone surgical treatment via VATS. A higher proportion of early‐stage patients treated via surgery were underwent adjuvant radiotherapy, whereas advanced‐stage patients were more likely to have undergone chemoradiotherapy. A total of 45 patients (57%) received chemotherapy, of which platinum drugs plus paclitaxel was the most frequently used, being administered in 38 patients (48.1%). Other chemotherapy treatments included cisplatin plus doxorubicin plus cyclophosphamide (*n* = 3), cisplatin plus doxorubicin plus vincristine plus cyclophosphamide (*n* = 2), and gemcitabine plus cisplatin (*n* = 2).

**TABLE 2:** Clinical characteristics in individuals with different stages of disease.

Features	Early‐stage	Advanced‐stage	*p*
Sex, *n* (%)			0.769
Female	14 (42.4%)	18 (39.1%)	
Male	19 (57.6%)	28 (60.9%)	
Age, years			
Mean ± SD	56.2 ± 11.2	53.5 ± 10.2	0.267
Year of diagnosis			0.436
2008–2014	15 (45.5%)	25 (54.3%)	
2015–2020	18 (54.5%)	21 (45.7%)	
Tumor size, cm			<0.001
≤4.6	25 (75.8%)	15 (32.6%)	
>4.6	8 (24.2%)	31 (67.4%)	
Invasive treatment			0.001
Biopsy	0 (0.0%)	12 (26.1%)	
Surgery	33 (100%)	34 (73.9%)	
Surgery approach^a^			<0.001
VATS	21 (63.6%)	7 (20.6%)	
Thoracotomy	12 (36.4%)	27 (79.4%)	
Resection extent^a^			<0.001
Complete resection	32 (97%)	18 (52.9%)	
Incomplete resection	1 (3%)	16 (47.1%)	
Postoperative adjuvant therapy^a^			0.008
Radiotherapy alone	19 (57.6%)	10 (29.4%)	
Chemotherapy alone	4 (12.1%)	8 (23.5%)	
Chemoradiation	5 (15.2%)	15 (44.1%)	
None	5 (15.2%)	1 (2.9%)	

^a^ Surgical approach, resection extent, and postoperative adjuvant therapy were only statistically analyzed in surgical patients (*n* = 67).

### 3.2 Survival analysis

The median OS of the overall patient cohort in this study was 99 months (95% confidence interval [CI], 42.3–155.7), with respective 5‐ and 10‐ year OS rates of 65.5% and 49.4%. The median PFS of the overall patient cohort was 62 months (95% CI, 37.0–87.0), with respective 5‐ and 10‐ year PFS rates of 52.3% and 37.9%.

**FIGURE 4:**
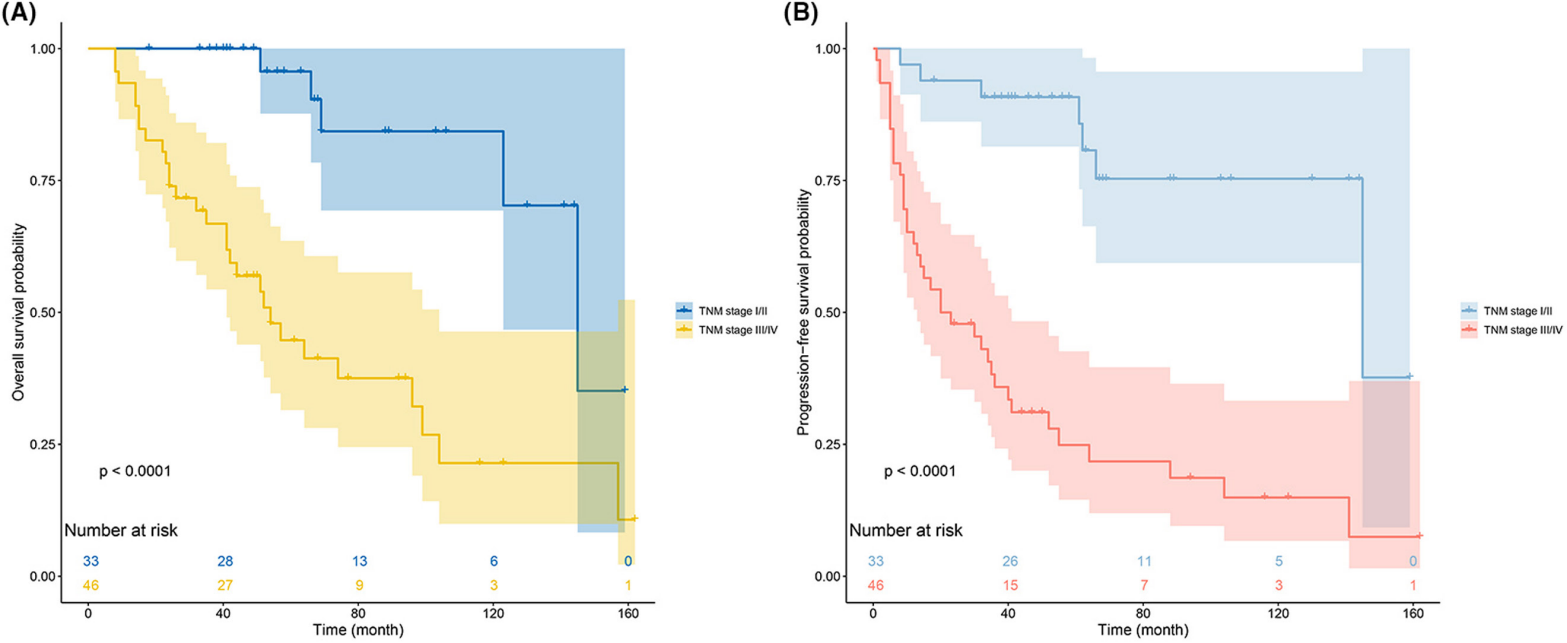
OS (A) and PFS (B) outcomes in different stage subgroups.

Kaplan–Meier curve analyses revealed that advanced disease was associated with significant reductions in patient OS and PFS (median OS: 145 months [95% CI, 113.2–176.8] vs. 54 months [95% CI, 37.4–70.6], *p* < 0.001; median PFS: 145 months [95% CI, 33.9–256.1] vs. 20 months [95% CI, 1.8–38.2], *p* < 0.001) (Figure 4).

**FIGURE 5:**
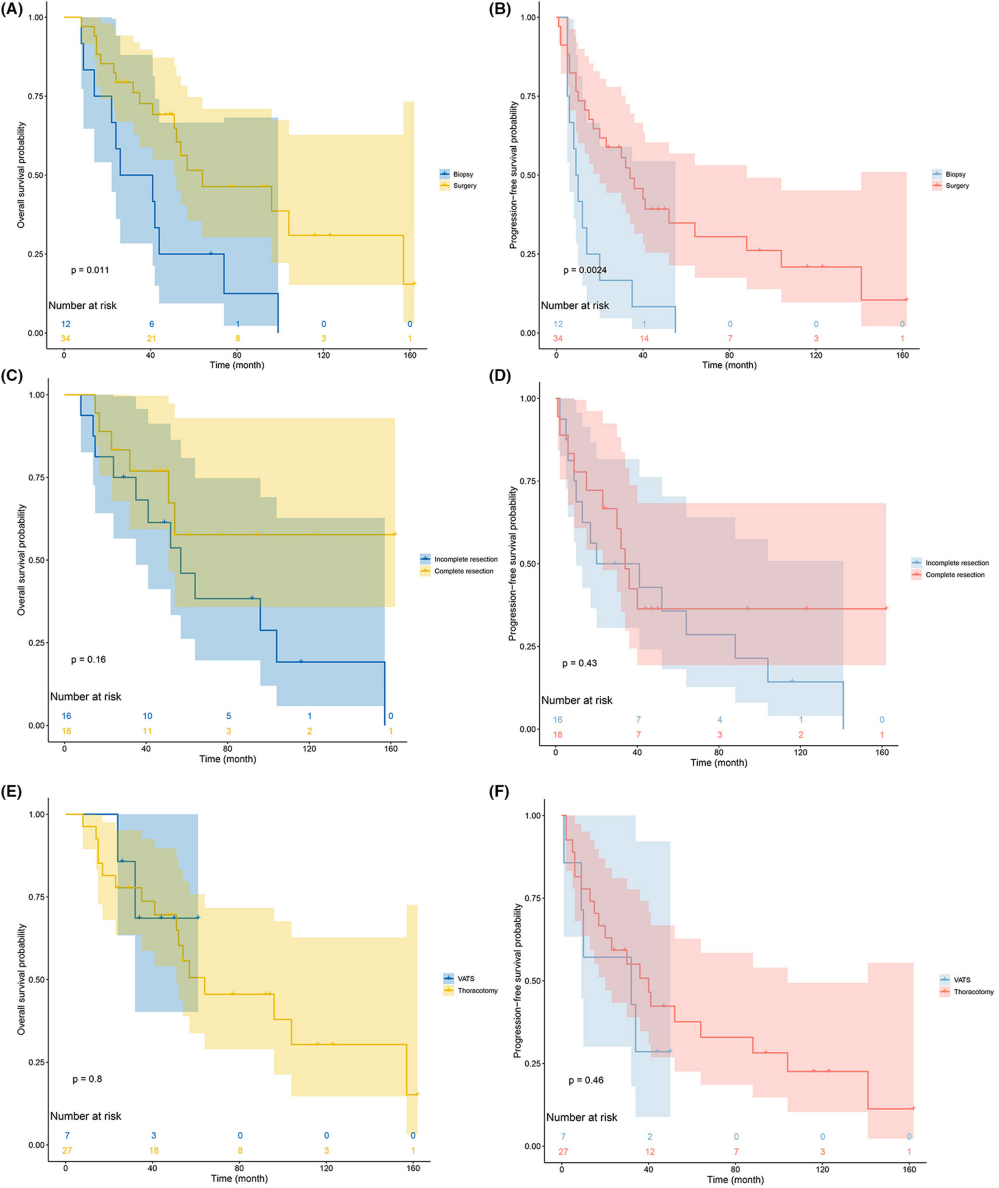
Kaplan–Meier curve analyses of survival outcomes in advanced‐stage patients subjected to different treatment regimens. (A, B) Survival curves corresponding to patients that had undergone surgery or biopsy; (A) OS, (B) PFS. (C, D) Survival curves for patients that had undergone surgery with complete or incomplete tumor resection; (C) OS, (D) PFS. (E, F) Survival curves corresponding to patients that underwent surgery via different approaches; (E) OS, (F) PFS.

For individuals with early‐stage disease, adjuvant therapy was not associated with any improvement in OS (*p* = 0.354) or PFS (*p* = 0.268). No differences in patient survival outcomes were identified as a function of the surgical approach (OS: *p* = 0.444, PFS: *p* = 0.725), the extent of tumor resection (OS: *p* = 0.820, PFS: *p* = 0.754), or the specific form of adjuvant therapy employed (OS: *p* = 0.812, PFS: *p* = 0.133). Surgical treatment was associated with improved OS (*p* = 0.011) and PFS (*p* = 0.002) in individuals with advanced‐stage disease. Among patients that had undergone surgery, adjuvant therapy was associated with an improved prognosis (OS: *p* = 0.029, PFS: *p* = 0.002). There was no significant impact of the extent of resection (OS: *p* = 0.214, PFS: *p* = 0.425), surgical approach (OS: *p* = 0.798, PFS: *p* = 0.463), or adjuvant therapy modality (OS: *p* = 0.313, PFS: *p* = 0.471) on patient outcomes (Figure 5).

### 3.3 Prognostic factors in TSCC patients

The multivariate analysis showed that advanced TNM stage and not having undergone surgical treatment were associated with worse OS and PFS outcomes in the overall patient cohort (Table 3).

**TABLE 3:** Univariate and multivariate analysis of OS and PFS in the overall patient cohort.

	Univariate analysis on OS		Multivariate analysis on OS		Univariate analysis on PFS		Multivariate analysis on PFS	
	HR (95% CI)	*p*	HR (95% CI)	*p*	HR (95% CI)	*p*	HR (95% CI)	*p*
Age	1.006 (0.973–1.041)	0.715			0.999 (0.971–1.028)	0.958		
Sex								
Female	Reference				Reference			
Male	1.251 (0.604–2.592)	0.542			0.962 (0.526–1.760)	0.901		
Year of diagnosis								
2008–2014	Reference				Reference			
2015–2020	0.788 (0.353–1.758)	0.560			0.933 (0.492–1.770)	0.831		
Tumor size	1.132 (0.963–1.332)	0.134	0.968 (0.782–1.198)	0.764	1.149 (1.017–1.299)	0.026	0.960 (0.807–1.141)	0.642
≤4.6 cm	Reference		Reference		Reference		Reference	
>4.6 cm	1.706 (0.846–3.437)	0.135	0.971 (0.459–2.055)	0.939	1.704 (0.931–3.118)	0.084	0.720 (0.373–1.390)	0.328
TNM stage								
I/II	Reference		Reference		Reference		Reference	
III/IV	5.833 (2.242–15.177)	<0.001	4.676 (1.555–14.062)	0.006	6.761 (2.992–15.273)	<0.001	5.978 (2.381–15.007)	<0.001
Treatment								
Biopsy	Reference		Reference		Reference		Reference	
Surgery	0.198 (0.094–0.418)	<0.001	0.350 (0.160–0.764)	0.008	0.170 (0.083–0.351)	<0.001	0.310 (0.148–0.650)	0.002

In individuals with early‐stage disease, no variables were significantly associated with patient OS or PFS in the univariate or multivariate analyses (Table S1). Only the adjuvant therapy approach was eligible for incorporation into multivariate analysis for patients with advanced‐stage disease. The extent of resection was also assessed as a covariate. This showed that all forms of adjuvant therapy were independently predictive of longer PFS. In contrast, only adjuvant chemoradiotherapy was associated with increased OS (Table 4).

**TABLE 4:** Univariate and multivariate analysis of OS and PFS in advanced‐stage surgical patients.

Variable	Univariate analysis on OS		Multivariate analysis on OS		Univariate analysis on PFS		Multivariate analysis on PFS	
	HR (95% CI)	*p*	HR (95% CI)	*p*	HR (95% CI)	*p*	HR (95% CI)	*p*
Age	1.023 (0.975–1.074)	0.348			1.025 (0.985–1.067)	0.224		
Sex								
Female	Reference				Reference			
Male	1.058 (0.407–2.749)	0.907			1.115 (0.507–2.450)	0.787		
Year of diagnosis								
2008–2014	Reference				Reference			
2015–2020	0.731 (0.239–2.240)	0.584			1.275 (0.533–3.051)	0.586		
Tumor size								
≤4.6 cm	Reference				Reference			
>4.6 cm	0.658 (0.243–1.785)	0.411			0.642 (0.279–1.478)	0.298		
Surgery approach								
VATS	Reference				Reference			
Open thoracotomy	1.219 (0.267–5.568)	0.798			0.687 (0.249–1.895)	0.468		
Resection extent								
Complete resection	Reference		Reference		Reference		Reference	
Incomplete resection	1.866 (0.686–5.079)	0.222	2.105 (0.725–6.118)	0.171	1.379 (0.622–3.061)	0.429	1.527 (0.673–3.464)	0.311
Adjuvant therapy								
None	Reference		Reference		Reference			
Radiotherapy alone	0.138 (0.013–1.425)	0.096	0.094 (0.008–1.058)	0.056	0.063 (0.005–0.762)	0.03	0.051 (0.004–0.640)	0.021
Chemotherapy alone	0.209 (0.021–2.058)	0.18	0.134 (0.012–1.479)	0.101	0.085 (0.007–1.003)	0.05	0.067 (0.005–0.830)	0.035
Chemoradiation	0.084 (0.008–0.845)	0.035	0.056 (0.005–0.623)	0.019	0.046 (0.004–0.544)	0.015	0.036 (0.003–0.450)	0.010

## 4 DISCUSSION

The present study was undertaken to identify TSCC‐related features and prognostic factors through a single‐center analysis of 79 patients with this rare disease. Together, these results offer updated real‐world evidence that can guide the treatment and evaluation of this uncommon malignancy.

The analyses revealed that the TNM system was slightly superior to the Masaoka system when used to predict TSCC patient prognostic outcomes. Moreover, advanced TNM staging and not having undergone surgical treatment were independently associated with worse patient OS and PFS outcomes. No differences in patient survival outcomes were noted when comparing treatment via VATS or thoracotomy, or patients that underwent incomplete or complete tumor resection. Postoperative adjuvant radiotherapy, chemotherapy, and chemoradiotherapy were correlated with improved PFS in patients with advanced disease, but only chemoradiotherapy was independently associated with improved OS.

The primary characteristics of TSCC patients in our study were that most were male, diagnosed when middle‐aged, and exhibited an insidious disease onset with rare comorbidity of MG. These characteristics were consistent with those found in other studies on TSCC patients.^8^, ^12^, ^14^

The Masaoka system is the most commonly used tool when staging TETs. However, the use of this system in assessing thymic carcinoma remains controversial.^9^, ^12^, ^18^ For example, the single‐center study noted above^12^ found the Masaoka stage to be unrelated to TSCC patient OS or disease‐free survival (DFS), and similar results have also been reported in an analysis of thymic carcinoma.^9^ These outcomes demonstrated that the Masaoka system may be not appropriate for TSCC. Instead, since the proposal of the TNM system, several studies have found it was at least as effective as the Masaoka system, and also acted as a prognostic factor in thymic carcinoma, indicating its potential for use in TSCC.^19^ In the present study, we utilized time‐dependent ROC curves. We discovered that the TNM staging system had a slightly higher AUC value than the Masaoka system. In addition, the multivariate analysis supported the TNM staging system's independent prognostic importance.

Our study confirmed that surgical treatment is associated with better patient prognosis, even for individuals with advanced disease. However, data on the relevance of the extent of tumor resection remain somewhat controversial. The present study noted that complete resection was not an independent protective factor, in line with the results of Shen et al.^20^ On the contrary, the retrospective analysis of Zhao et al.^12^ and the SEER study performed by Yang et al.^8^ both found that complete resection was predictive of improved survival outcomes in TSCC patients. Some studies of thymic carcinoma patients have also identified complete resection as an important prognostic factor.^9^, ^10^, ^21^, ^22^, ^23^ Although the reasons for this disparity are unknown, we continue to recommend complete resection as the ultimate surgical goal whenever possible. When total resection is deemed impossible, surgery may still be advised for selected individuals.

The number of thoracic surgical procedures performed using the VATS approach has risen in recent years. Nevertheless, few studies have compared outcomes associated with VATS and thoracotomy procedures in TSCC patients. In the present analysis, there were no differences in OS or PFS between VATS and thoracotomy, and VATS was not linked with increased rates of incomplete resection (*p* = 0.09). Thus, VATS represents a promising choice for appropriate patients.

There is no consensus on the optimal postoperative adjuvant therapy regimen for treating TSCC. In the present study, we discovered that adjuvant radiotherapy and chemotherapy alone were associated only with extended PFS but not OS. Nevertheless, postoperative chemoradiotherapy was found to be an independent protective factor for both OS and PFS in patients with advanced‐stage TSCC. Several studies^5^, ^22^, ^24^, ^25^, ^26^ have observed better outcomes in patients who underwent postoperative radiotherapy, especially those with advanced disease. The effect of postoperative chemotherapy was unclear. The main view is that adjuvant chemotherapy may be of less significance than radiotherapy, which has been verified by some studies.^10^, ^27^ However, other studies have found that adjuvant chemotherapy could improve the prognosis of thymic carcinoma, especially in prolonging PFS.^12^, ^28^ Besides, Yang et al.^8^ in their SEER study indicated that TSCC may be more sensitive to chemotherapy than other histological types. Very few research studies on adjuvant therapeutic regimens have specifically investigated the effects of chemoradiotherapy treatment. Kim et al.^29^ found that adjuvant chemoradiotherapy was associated with a better prognosis in patients with Masaoka stage III thymic carcinoma. In conclusion, for advanced‐stage TSCC patients, adjuvant therapy should be administered, and chemoradiotherapy, in particular, should be considered in selected patients to improve both OS and PFS.

There are several limitations to this study. For instance, because this was a single‐center retrospective investigation, the results are prone to implicit bias. Furthermore, the sample size was small, necessitating further research with additional in‐depth subgroup analysis. Additionally, the 12‐year study period may have led to some differences in the treatment plans utilized. Besides, due to the unavailability of some data, treatment details, such as the dose and site of radiotherapy, were not recorded and compared.

## 5 CONCLUSIONS

These results suggest that the TNM staging system may be optimal for evaluating TSCC patients. Both advanced TNM stage and not having undergone surgical treatment were herein found to be independently associated with a risk of poorer OS and PFS outcomes. However, neither resection extent nor surgical approach was an independent factor of TSCC patient survival. Therefore, VATS might be a better option for selected patients. After evaluation, surgery should be the first alternative for individuals with advanced‐stage TSCC, and postoperative chemoradiotherapy should be given to improve patient outcomes. Further in‐depth studies with uniform treatment guidelines will be essential to gain a more detailed understanding regarding the prognosis of patients with this form of cancer.

## AUTHOR CONTRIBUTIONS

**Meng‐Xin Zhou:** Conceptualization (equal); data curation (equal); formal analysis (lead); investigation (equal); methodology (equal); validation (equal); visualization (equal); writing – original draft (lead). **Ye‐Ye Chen:** Conceptualization (equal); data curation (equal); formal analysis (equal); methodology (equal); project administration (equal); resources (equal); validation (equal); writing – original draft (equal); writing – review and editing (lead). **Cheng Huang:** Project administration (equal); resources (equal); validation (equal). **Lei Liu:** Formal analysis (equal); methodology (equal); resources (equal). **Gui‐Ge Wang:** Resources (equal); visualization (equal). **Jia‐Qi Zhang:** Investigation (equal). **Wen‐Liang Bai:** Investigation (equal). **Ke Zhao:** Formal analysis (equal); methodology (equal). **Shan‐Qing Li:** Conceptualization (equal); data curation (equal); project administration (lead); resources (equal); supervision (lead); validation (equal); writing – review and editing (equal).

## FUNDING INFORMATION

This work did not receive any specific grant from funding agencies in the public, commercial, or not‐for‐profit sectors.

## CONFLICT OF INTEREST STATEMENT

The authors declare that the research was conducted in the absence of any commercial or financial relationships that could be construed as a potential conflict of interest.

## ETHICS APPROVAL STATEMENT

The institutional review board of Peking Union Medical College Hospital has approved the present study (ZS‐3305). All involved patients in the present study had provided consents for the research use of their data at the time of hospital admission. The study was thus exempt from the further need to obtain informed patient consent.

## CLINICAL TRIAL REGISTRATION NUMBER (IF APPLICABLE).

The present is not registered.

## Supporting information


Table S1.

## Data Availability

The datasets developed and used in the present research are not publicly accessible, but they are available from the corresponding author upon reasonable request.
