# Supplementary material for: Identification of predictors of long‐term survival and prognostic outcomes in thymic squamous cell carcinoma: A real‐world study
Source: Cancer Med. 2023 Jun 17;12(13):14025–36. doi: 10.1002/cam4.6049 (PMC10358255; doi:10.1002/cam4.6049)
Supplement: Supplementary file 1 — Table S1. [file CAM4-12-14025-s001.docx]

| **Supplement Table. Univariate analysis of OS and PFS in early-stage patients** | | | | | |
| --- | --- | --- | --- | --- | --- |
| Variable | OS | | PFS | |  |
|  | HR (95% CI) | p | HR (95% CI) | p |  |
| Age | 1.028 (0.937-1.128) | 0.560 | 0.994 (0.922-1.071) | 0.869 |  |
| Sex |  |  |  |  |  |
| Female | Reference |  | Reference |  |  |
| Male | 0.271 (0.025-3.002) | 0.287 | 0.256 (0.046-1.420) | 0.119 |  |
| Year of diagnosis |  |  |  |  |  |
| 2008~2014 | Reference |  | Reference |  |  |
| 2015~2020 | 1.674 (0.142-19.742) | 0.682 | 0.722 (0.123-4.249) | 0.719 |  |
| Tumor size |  |  |  |  |  |
| ≤4.6 cm | Reference |  | Reference |  |  |
| ＞4.6 cm | 0.723 (0.075-6.983) | 0.779 | 1.159 (0.209-6.438) | 0.866 |  |
| Surgery approach |  |  |  |  |  |
| VATS | Reference |  | Reference |  |  |
| Open thoracotomy | 2.421 (0.236-24.808) | 0.457 | 1.336 (0.265-6.744) | 0.726 |  |
| Resection extent |  |  |  |  |  |
| Complete resection | Reference |  | Reference |  |  |
| Incomplete resection | 1.336 (0.109-16.351) | 0.821 | 1.491 (0.122-18.254) | 0.755 |  |
| Adjuvant therapy |  |  |  |  |  |
| None | Reference |  | Reference |  |  |
| Radiotherapy only |  | 0.956 |  | 0.962 |  |
| Chemotherapy only |  | 0.955 |  | 0.956 |  |
| Chemoradiation |  | 0.957 |  | 0.958 |  |
